# The public’s attitude to and acceptance of periodic doses of the COVID-19 vaccine: A survey from Jordan

**DOI:** 10.1371/journal.pone.0271625

**Published:** 2022-07-20

**Authors:** Sawsan Abuhammad, Omar F. Khabour, Karem H. Alzoubi, Shaher Hamaideh, Baker A. Alzoubi, Waed S. Telfah, Farah K. El-zubi

**Affiliations:** 1 Department of Maternal and Child Health, Jordan University of Science and Technology, Irbid, Jordan; 2 Dept. of Medical Laboratory Sciences, Jordan University of Science and Technology, Irbid, Jordan; 3 Department of Pharmacy Practice and Pharmacotherapeutics, University of Sharjah, Sharjah, UAE; 4 Department of Clinical Pharmacy, Jordan University of Science and Technology, Irbid, Jordan; 5 Department of Community and Mental Health Nursing, Faculty of Nursing, The Hashemite University, Zarqa, Jordan; 6 Faculty of Medicine, Jordan University of Science and Technology, Irbid, Jordan; Universitas Syiah Kuala, INDONESIA

## Abstract

**Aims:**

This study surveyed people regarding their acceptance of periodic doses (i.e., annual boosters) of the COVID-19 vaccine. Moreover, factors that correlate with attitudes toward periodic COVID-19 vaccines were assessed and identified.

**Method:**

The study employed a cross-sectional methodology. The study questionnaire was distributed using Google Forms. Data were collected during the last quarter of 2021, and 1,416 adults (18 years old and over) from Jordan responded. Acceptance of COVID-19 periodic vaccine doses was calculated as a percentage of the total number of study participants, and their attitudes were scored. A multiple regression model was used to determine the predictors of public attitudes toward the annual dose of COVID-19 vaccines.

**Results:**

The acceptance rate for receiving periodic doses of the COVID-19 vaccine was low (19.3%). Additionally, 26% of participants were unsure about receiving additional doses of the vaccine. However, 54.7% had a negative attitude toward getting periodic doses. The mean score for attitudes toward periodic doses was 47.9 (range: 29–66). Among the identified factors leading to decisions not to receive periodic doses were side effects (49.1%), waiting for further clinical studies (38.8%), and perceived no risk of contracting COVID-19 (17.7%). Regression analysis showed that income, educational attainment, and following the news about COVID-19 were predictors of participants’ attitudes toward the periodic COVID-19 vaccine.

**Conclusion:**

Acceptance of periodic doses of the COVID-19 vaccine in Jordan is low, and the public’s attitude is generally negative. Health programs and educational interventions are needed to promote vaccine acceptance and positive attitudes.

## Introduction

The COVID-19 pandemic is continuing to overwhelm global populations with significant consequences for global health and the economy. Vaccination is the main preventative measure that can help control and mitigate the spread of serious infectious diseases [1, 2]. A large study based on COVID-19 vaccine information conducted by Our World in Data showed that new cases and new deaths per million people gradually decreased as the rate of vaccination coverage increased [3]. Other studies reported similar conclusions, indicating a correlation between vaccination and better COVID-19 outcomes or a lower number of new cases [4, 5]. Still, some studies, e.g., [6] have shown no such correlations. Therefore, the implementation of an effective vaccine program against COVID-19 is essential for overcoming the pandemic [7–9]. Such a program can be implemented through collaboration between governments, scientific communities, and pharmaceutical manufacturers [10, 11].

The World Health Organization (WHO) authorized COVID-19 vaccines under the emergency license [12]. COVID-19 vaccines are effective against severe illness, hospitalization, and death resulting from infection with different strains of SARS-CoV-2. The manufacturers of COVID-19 vaccines use antibody levels as surrogate biomarkers to determine vaccine efficacy. As is the case following most vaccinations, COVID-19 antibody levels slowly decline after vaccinations [13–16]. For example, a previous study conducted on 605 adults who received two doses of the Pfizer-BioNTech or Oxford-AstraZeneca vaccines showed a significant trend of declining antibody levels over time [14]. In another study conducted on other mRNA vaccines, a decrease in neutralizing antibodies was reported three months after the second dose [16]. Due to these vaccines’ low efficacy in increasing and maintaining the number of neutralizing antibodies, individuals vaccinated with inactivated SARS-CoV-2 (such as Sinovac-CoronaVac) vaccines should receive booster doses of a heterologous vaccine [17]. In general, there is agreement that individuals who have received two doses of COVID-19 vaccines should receive additional doses periodically [18]. Such additional doses have been shown to be beneficial and safe [19–22]. Additional doses are also strongly recommended for adults with underlying health conditions living in long-term care and for those living in high-risk environments [23, 24]. However, the benefits of frequent doses of vaccines remain debatable. In a study conducted on 96 heart transplant patients, the third dose of the COVID-19 vaccine elicited strong humoral and cellular immune responses with a good safety profile [25]. Similarly, repeated vaccinations against SARS-CoV-2 have been shown to elicit a robust polyfunctional T cell response in allogeneic stem cell transplantation recipients [26]. However, a study conducted on repeated influenza vaccinations showed a significant negative effect on the immune response [27]. This created some fear that periodic COVID-19 vaccine doses could negatively impact the immune response and may not be practical. Therefore, in the present study, the acceptance of periodic doses (i.e., annual boosters) of the COVID-19 vaccine was surveyed. Moreover, attitudes toward periodic COVID-19 vaccinations and these attitudes’ predictive factors were assessed.

## Method

### Study design, population, and sampling

This study was conducted from October through November 2021. A cross-sectional study design that adopted a convenience sampling procedure was used. An anonymous survey was distributed online using Google Forms; the survey link was posted on major social media platforms, such as Facebook and WhatsApp. Inclusion criteria were being an adult (≥ 18 years old) and living in Jordan during the study. The study’s sample size was determined using G*Power, version 3.1., Universitat Kiel, Germany, based on convenience sampling, an alpha of 0.05, a small effect size, and a power of 0.95. The minimum number of subjects required was 1410. The survey was attempted (started) by 1555 participants, and only 1416 completed the survey to the final stage, representing the final working number of study subjects. The population of Jordan is about 11 million, and most are Muslims and of Arab ethnicity.

### Measures

The study’s questionnaire was self-administered. It was created based on past frameworks and studies that evaluated attitudes toward vaccines for novel contagious infections, such as COVID-19, Ebola, and H1N1 [28]. The questionnaire had three parts and a cover page. The first part included sociodemographic characteristics, such as marital status, age, smoking, educational attainment, employment status, health status, and family income. The second part required participants to answer a question about whether they agreed to receive periodic doses of the COVID-19 vaccine. The third part included 24 items related to their attitudes toward COVID-19 vaccination. The items were presented to the respondents as categorical variables and assessed using a five-point Likert scale. The responses ranged from strongly disagree (1) to strongly agree (5) for items that yielded positive attitude points (Table 2, items without *), and from strongly disagree (5) to strongly agree (1) for items that yielded negative attitude points (Table 2, items with *). The range of scores was between 24 and 120. Thus, when the total score was higher, this meant a more positive attitude toward receiving a periodic dose of the vaccine. The fourth part included questions related to the probability of contracting COVID-19, vaccine benefits, and barriers to receiving the periodic vaccine. The Cronbach’s alpha for the attitude parts of the instrument was 0.89.

### Ethical considerations

This study was approved by the Institutional Review Board of Hashemite University (approval ID: 11/1/2021-2022). Participation in the study was completely voluntary, and electronic consent was obtained from all participants in the form of a required item in which the “agree” choice was mandatory before participants obtained access to the questionnaire. Participants were also given the choice to skip any questions they did not want to answer. The cover page provided adequate information on the study so that participants could make informed, voluntary, and rational decisions regarding their participation. To ensure privacy, no personal information was included in the questionnaire.

### Data analysis

The authors used the Statistical Package for Social Sciences (SPSS) version 25 to analyze the data. Descriptive statistics were used to define the sample characteristics. Custom tables were used to describe people’s responses toward periodic COVID-19 vaccine doses. A multiple regression test was used to determine the predictors of the public’s attitudes toward annual doses of the COVID-19 vaccine.

## Results

### Demographic characteristics

A total of 1416 participants completed the online questionnaire, meaning it had a 91.1% completion rate (Table 1). The mean age was 31.9 years (standard deviation [SD] = 9.3). The number of females was 958 (67.7%), and the number of males was 458 (32.3%). Almost 1045 (73.8%) had health insurance. Approximately 50% of the sample were either students or had no jobs. The majority of participants were educated, had a low income (<400 JD), were married, had children, lived in a city, were non-smokers, and had not received the influenza vaccination (Table 1).

**Table 1:** Demographical characteristics for the participants (N = 1416).

Variable	Categories	N	%
**Gender**	Male	458	32.3
	Female	958	67.7
**Working status**	I do not work (including students)	709	50.0
	Full-time	559	39.5
	Part-time	148	10.5
**Income level**	Less than 400 Jordanian dinars	814	57.5
	401 to 800	468	33.1
	801 to 1500	96	6.8
	More than 1500	38	2.7
**Educational attainment**	High school or lower	442	31.2
	Diploma	154	10.9
	Graduate	627	44.3
	Postgraduate	193	13.6
**Marital status**	Unmarried	608	42.9
	Married	773	54.6
	Divorced	35	2.5
**Children**	No	690	48.7
	Yes	715	50.5
**Living area**	Urban	905	63.9
	Rural	511	36.1
**Smoking**	No	870	61.4
	Yes	546	38.6
**Health insurance**	No	371	26.2
	Yes	1045	73.8
**Do you have a family member 65 years of age or older?**	No	743	52.5
	Yes	673	47.5
		
		
**How often do you see news related to COVID-19?**	Never	343	24.2
	Rarely	387	27.3
	Sometimes	348	24.6
	Always		
**Did you have the flu vaccination?**	No	860	60.7
	Yes	426	30.1
	Maybe	130	9.2

### Public acceptance of periodic doses of the COVID-19 vaccine

The results showed that 273 (19.3%) participants agreed to receive periodic doses of the COVID-19 vaccine. In addition, 368 (26.0%) were unsure about receiving periodic vaccinations. However, 775 (54.7%) did not want periodic doses of the vaccine. For males, 114 (24.9%) wanted periodic doses of the COVID-19 vaccine, whereas this number was 159 (16.6%) for females.

### Description of people’s attitudes toward periodic doses of the COVID-19 vaccine

The mean score for attitudes toward periodic doses of the COVID-19 vaccine among Jordanians was 47.96 ± SD = 9.2. The range of scores was between 29 and 66. The mean score indicated that Jordanians held a negative attitude toward getting periodic doses of the vaccine (Table 2). The highest numbers of “agree” responses were for the following items: “In general, vaccination is a good thing” (547, 38.6%), “It would be very easy for me to get the periodic doses of the COVID-19 vaccine” (466, 32.9%), and “If a healthcare professional recommends periodic doses of the COVID-19 vaccine, I will get vaccinated” (441, 31.2%). However, the highest numbers of “disagree” responses were for the following statements: “The periodic doses of the COVID-19 vaccine must be mandatory for every person able to receive it” (671, 47.4%), “I’m afraid of needles” (700, 49.4%), and “If I get periodic doses, I think I will not need to follow the social distancing and other restrictions imposed as a result of the coronavirus” (619, 43.7%; Table 2).

**Table 2:** Description of Jordanians’ attitudes toward receiving periodic doses of COVID-19 vaccine.

	Disagree		Neutral		Agree	
	Count	%	Count	%	Count	%
1. Periodic COVID-19 vaccinations must be made mandatory for every person who is able to receive them.	671	47.4	479	33.8	266	18.8
2. Without a COVID-19 periodic vaccination, I would probably have contracted COVID-19.	521	36.8	587	41.5	308	21.8
3. The periodic COVID-19 vaccination will protect me from COVID-19.	565	39.9	567	40.0	284	20.1
4. If I do not get the periodic COVID-19 vaccination and I get infected with COVID-19, I will regret not getting vaccinated.	566	40.0	495	35.0	355	25.1
5. It would be very easy for me to get the periodic doses of the COVID-19 vaccine.	370	26.1	580	41.0	466	32.9
6. The periodic COVID-19 vaccination may infect me with the virus.*	522	36.9	621	43.9	273	19.3
7. I would be worried about suffering from the side effects of the periodic dose of COVID-19 vaccination.*	366	25.8	482	34.0	568	40.1
8. I may regret receiving periodic doses of the COVID-19 vaccine if I later experience side effects from the vaccination.*	422	29.8	503	35.5	491	34.7
9. The periodic COVID-19 vaccination would be too new for me to be confident about getting vaccinated.*	402	28.4	613	43.3	401	28.3
10. Most people will receive periodic doses of the COVID-19 vaccine.	451	31.9	639	45.1	326	23.0
11. Other people like me will receive periodic doses of the COVID-19 vaccine.	419	29.6	681	48.1	316	22.3
12. In general, vaccination is a good thing.	356	25.1	513	36.2	547	38.6
13. I am afraid of needles.*	700	49.4	428	30.2	288	20.3
14. If I get periodic doses, I think I will not need to follow the social distancing and other restrictions imposed due to COVID-19.*	619	43.7	506	35.7	291	20.6
15. I know enough about COVID-19 to make an informed decision about whether to get a periodic vaccination.	374	26.4	607	42.9	435	30.7
16. I know enough about the COVID-19 vaccines to make an informed decision about whether to get vaccinated.	409	28.9	622	43.9	385	27.2
17. Only people at risk of serious illness from COVID-19 need a periodic dose of vaccination.*	633	44.7	536	37.9	247	17.4
18. My family will approve periodic doses for the COVID-19 vaccination.	438	30.9	632	44.6	346	24.4
19. My friends will approve periodic doses for the COVID-19 vaccination.	434	30.6	735	51.9	247	17.4
20. If the government recommends periodic vaccinations for COVID-19, I will get vaccinated.	455	32.1	555	39.2	406	28.7
21. If a healthcare professional recommends periodic vaccination for COVID-19, I will get vaccinated.	432	30.5	542	38.3	442	31.2
22. The periodic COVID-19 vaccination is just a way for vaccine manufacturers to make money.*	447	31.6	676	47.7	293	20.7
23. The periodic COVID-19 vaccination will allow us to return to normal life.	404	28.5	652	46.0	360	25.4
24. There will be no point in getting periodic doses against COVID-19 unless I can return to my normal life.*	426	30.1	607	42.9	383	27.0

* Indicates points that represent negative attitudes toward COVID-19 vaccination, where they were scored in reverse compared to other points listed.

### Responses of the participants to the benefits and barriers of periodic doses of the COVID-19 vaccine

The main motivations encouraging participants to agree to periodic doses of the COVID-19 vaccine were as follows: “Periodic doses will lower the chance of getting COVID-19 disease” (62.2), “Periodic doses will protect my family from the COVID-19 virus and its consequences” (51.4%), “Periodic doses will protect my job” (34.2%), and “Periodic doses will decrease the expenses of hospitalization” (28.6%). The main barriers to receiving periodic doses of the COVID-19 vaccine were fear of unexpected side effects (49.1%), waiting for more results (38.8%), and perceived no risk of contracting the COVID-19 virus (17.7%).

### Predictors of attitude toward periodic doses of the COVID-19 vaccine

The regression model was significant (F = 9.02, P = 0.001), which means that some factors could predict Jordanians’ attitudes toward periodic doses of the COVID-19 vaccine. These factors were income (B = 0.132, P < 0.000), educational attainment (B = -0.07, P = 0.011), suffering from side effects from the previous vaccine doses (B = -0.079, p = .011), and hearing news (B = 0.07, P = 0.004). These results indicate that those with a higher income, those with high educational attainment, and those who follow the news about COVID-19 had more positive attitudes toward receiving periodic doses of the vaccine (Table 3). However, other demographic factors, such as having health insurance, smoking, and having a job, were not associated with acceptance of periodic doses (P > 0.05).

**Table 3:** Predictors of attitudes toward receiving periodic doses of the COVID-19 vaccine.

Model		Unstandardized Coefficients		Standardized Coefficients	t	Sig.
		B	Std. Error	Beta		
	(Constant)	37.159	2.003		18.547	0.000
	Age	0.038	0.026	0.054	1.466	0.143
	Gender	-0.692	0.425	-0.047	-1.628	0.104
	Having a job	0.099	0.146	0.021	0.678	0.498
	Income	1.241	0.267	0.132	4.651	0.000
	Educational attainment	-0.375	0.148	-0.070	-2.534	0.011
	Marital status	0.426	0.568	0.033	0.750	0.454
	Children	0.346	0.641	0.025	0.540	0.589
	Smoking	0.689	0.397	0.048	1.736	0.083
	Heath insurance	-0.205	0.424	-0.013	-0.484	0.628
	Do you have a family member 65 years of age or older?	0.384	0.362	0.028	1.061	0.289
	Have you had COVID-19?	-0.181	0.192	-0.025	-0.942	0.346
	How often do you see news related to COVID-19?	0.482	0.167	0.076	2.880	0.004
	Did you receive the flu vaccine?	0.503	0.282	0.048	1.782	0.075

## Discussion

This study found that the Jordanian public has a low acceptance of periodic doses of the COVID-19 vaccine. About a quarter of the participants showed a willingness to receive periodic doses. Currently, several countries, including Jordan, recommend additional doses for the general population and for individuals at risk of contracting a severe form of the disease. Unfortunately, countries in the Middle East/North African region still suffer from a lack of vaccine acceptance [29]. For example, in a study conducted in Algeria, the acceptance rate of additional doses was only 51.6% [30]. A similar acceptance rate (55.3%) was reported in Saudi Arabia [31]. However, higher acceptance rates have been reported in developed countries, such as Japan (97.7%), China (90%), and Italy (85.7%) [32–34]. In addition, moderate acceptance of booster doses was reported in the United States (62%), the Czech Republic (71.3%), and Poland (70%) [35–37]. Thus, more efforts should be made in Jordan and other developing countries to enhance vaccine acceptance and overcome the barriers associated with vaccine hesitancy.

The current study can benefit from the literature that examined the acceptance of annual vaccination programs against seasonal influenza [38]. In studies conducted in developing countries, the acceptance rate for influenza vaccinations was reported to be about 52% in Oman, 58% in Saudi Arabia, and 40.4% in Lebanon [39–41]. In the United States, about one-quarter of the population was reported to be hesitant to receive the annual influenza vaccines [42]. In Italy, 67.7% of master’s degree students in nursing and midwifery reported a willingness to receive the annual influenza vaccine [43]. Thus, vaccine acceptance rates seem to be higher in developed countries than in developing countries. Factors associated with annual influenza hesitancy were mainly safety and effectiveness issues [38, 44]. Thus, developing countries would benefit from the experience of developed countries in promoting vaccine acceptance among their populations.

Based on the literature, additional doses of the COVID-19 vaccine after the two initial doses are projected to be highly efficient (>90%) in averting COVID-19-related hospital admissions and serious illnesses compared to the two-dose program [45–47]. Additionally, the third dose has been estimated to be highly effective (>70%) in preventing COVID-19-related deaths compared to only two doses. Moreover, booster doses have also been shown to enhance the body’s immunity against different SARS-CoV-2 variants [48, 49]. Vaccine dose efficacy was similar in men and women among the different comorbidity groups [47]. The current study showed that the majority of the participants believed that periodic vaccine doses might decrease the chance of contracting COVID-19 and its consequences, and thus protect their jobs. In comparison, a study from Romania found that more than 85% of participants agreed that additional doses of the vaccine would protect them and their families against the severe consequences of COVID-19 [45]. Highlighting the benefits of booster doses in preventing severe forms of COVID-19 is expected to enhance people’s acceptance of periodic vaccinations.

The current study found that the main barriers to accepting periodic vaccine doses were fearing unexpected side effects, waiting for more clinical data, and not being at risk of contracting the COVID-19 virus. Safety concerns surrounding COVID-19 vaccines have been reported to affect the public’s perceptions in some previous studies [50, 51]. In fact, according to a multinational study, at the same level of hypothetical COVID-19 vaccine efficacy, side effects cause a huge decline in the vaccine acceptance rate [29]. The efficacy of COVID-19 vaccines was also previously reported as a barrier to accepting additional doses [52–54]. In Poland, almost 30% of the public refused any additional doses of the COVID-19 vaccine, with side effects being the main barrier [32]. It is worth mentioning that people in previous studies generally showed a more positive attitude toward additional doses of the Pfizer vaccine and mRNA vaccines than other types of vaccines that showed complications, such as clotting and thrombocytopenia [55, 56].

The present results showed that multiple factors predict people’s attitudes toward periodic doses of the COVID-19 vaccine. A positive attitude was found to be associated with higher income, high educational attainment, and frequent hearing of news about COVID-19. Other factors, such as gender, getting the flu vaccine, smoking, having health insurance, and having children, were not associated with attitudes toward periodic COVID-19 vaccination. In a previous study, attitudes toward receiving additional doses were not different when the gender factor was considered [32]. However, two studies showed that women were more compliant with protective measures and health policies during the COVID-19 pandemic [57, 58]. Yet another study suggested that in the younger population, women are more hesitant toward the COVID-19 vaccine [59]. In addition, a previous study showed that people who received the flu vaccine were more likely to receive additional doses of the COVID-19 vaccine [32]. With respect to educational attainment, several studies reported negative attitudes toward COVID-19 vaccination among people with low levels of education [60–62], as they could be misinformed about the effectiveness/side effects of the vaccines [11]. Regarding smoking and health insurance, this study expected that individuals without health insurance would be more positive toward vaccination to avoid healthcare costs if they were infected with a severe form of the disease [63, 64]. However, none of these factors were associated with vaccine acceptance in Jordan. The identification of different factors that might impact the public’s attitudes toward COVID-19 vaccination can help policymakers establish effective interventions that will help widen the acceptance of vaccination.

### Implications

The findings of this study may help decision makers and the health sector understand the public’s attitudes toward periodic doses of the COVID-19 vaccine. This understanding can be used in interventions to increase vaccination acceptance and enhance humoral immunity in the community. Additionally, identifying factors related to vaccination acceptance among the public has significant implications for building a positive attitude toward the COVID-19 vaccine. Furthermore, the literature suggests that appropriate public education can enhance vaccine acceptance.

### Limitations

The current study has some limitations. First, the study was conducted over a short period (about two months). Since the vaccine acceptance rate may be affected by waves of virus spread, it is recommended that the current results be confirmed by studies with longer durations. In addition, data were collected using self-reporting. Therefore, self-report bias cannot be ruled out. The study sample had more females than males, which could have an impact on the results obtained. Finally, the current study examined only attitudes toward periodic vaccination. The literature would benefit from investigations into the knowledge and practices regarding periodic COVID-19 vaccinations.

## Conclusions

The results showed that Jordanians have a negative attitude and low acceptance toward receiving periodic doses of the COVID-19 vaccine. Attitude was found to be affected by income, educational attainment, and whether the participants followed news about COVID-19. Among the identified barriers to accepting periodic doses were vaccine side effects, waiting for more clinical data, and perceived no risk of contracting COVID-19. The results can be utilized by health policymakers to enhance the acceptance of COVID-19 vaccines among the public.

## Supporting information

S1 Data (XLSX)
